# Influence of Prostate Volume on Targeted Biopsy Outcomes and PI-RADS Predictive Value for Significant Prostate Cancer

**DOI:** 10.3390/jcm14238476

**Published:** 2025-11-29

**Authors:** Shir Tiger, Igal Shpunt, Ilia Beberashvili, Yuval Avda, Vadim Smolyakov, Dmitry Lerman, Gal Goldshtein, Wael Shahabri, Dor Rubinshtein, Morad Jaber, Roy Croock, Adam Abu Marsa, Yaniv Shilo, Jonathan Modai, Dan Leibovici

**Affiliations:** 1 Department of Urology, Kaplan Medical Center, The Faculty of Medicine, The Hebrew University of Jerusalem, Rehovot 9190501, Israelroycr@clalit.org.il (R.C.); danle1@clalit.org.il (D.L.); 2 Department of Urology, Rabin Medical Center, Petach Tikva 4941492, Israel; 3 Department of Nephrology, Asaf Harofe Medical Center, Beer Yakov 7033001, Israel; iliabeberashvili63@gmail.com; 4 Department of Urology, Assuta Medical Center, Ashdod 7747629, Israel; yuvalavda@hotmail.com; 5 Department of Radiology, Kaplan Medical Center, The Faculty of Medicine, The Hebrew University of Jerusalem, Rehovot 9190501, Israel; bvsdrx@gmail.com (V.S.);; 6 Department of Urology, Laniado Medical Center, Nathanya 4244916, Israel

**Keywords:** prostate cancer, PIRADS, prostate volume, mpMRI, fusion biopsy, clinically significant prostate cancer, high-risk prostate cancer

## Abstract

**Background/Objectives**: Multiparametric MRI (mpMRI) and targeted biopsies have revolutionized prostate cancer (PC) detection through the Prostate Imaging Reporting and Data System (PIRADS). However, the effect of prostate volume on cancer detection and the predictive accuracy of PIRADS in the mpMRI-guided biopsy era remains unclear. The aim was to assess whether prostate volume affects detection rates of clinically significant prostate cancer (CSPC) and high-risk prostate cancer (HRPC) and modifies the predictive performance of the PIRADS score. **Methods**: We retrospectively analyzed 361 biopsy-naïve men who underwent mpMRI-fusion transperineal biopsies between 2016 and 2023. Lesions graded PIRADS ≥ 3 were targeted alongside systematic sampling. A receiver-operating characteristic (ROC) curve (AUC = 0.74) defined a 44 mL cutoff separating small (<44 mL; n = 160) and large (≥44 mL; n = 193) prostates. Logistic regression and cubic-spline analyses evaluated associations between prostate volume, PIRADS, and cancer outcomes. **Results**: Any cancer was detected in 74.3% of small versus 35.5% of large prostates (*p* < 0.001); CSPC in 42.5% vs. 19.6% (*p* < 0.001); HRPC in 14.3% vs. 5.5% (*p* < 0.001). Small prostate volume independently predicted any cancer (OR 7.31; 95% CI 4.22–12.7), CSPC (OR 5.08; 95% CI 2.87–8.99), and HRPC (OR 4.50; 95% CI 1.80–11.3). Between 40 and 70 mL, each 10 mL increase in volume reduced CSPC risk by 61% (*p* = 0.008). Prostate volume significantly modified PIRADS accuracy: in large glands, PIRADS 3 lesions carried only 2% risk for CSPC and 0% for HRPC, while in small prostates, PIRADS 3 conferred a 16.9-fold increased CSPC risk. **Conclusions**: Prostate volume inversely correlates with cancer detection and aggressiveness. PIRADS performance is volume-dependent; PIRADS 3 lesions in large prostates rarely represent significant cancer and may not warrant biopsy.

## 1. Introduction

Historically, prostate biopsies were guided by transrectal ultrasound (TRUS). The initial prostate biopsy protocol consisted of six-core (sextant) biopsies aimed at detecting prostate cancer. However, the detection rate of clinically significant prostate cancer (CSPC) was relatively low. By the early 1990s, an extended biopsy technique was introduced, incorporating 10–12 cores. This modification significantly increased the yield of clinically significant cancer detection, providing better gland coverage and improving diagnostic accuracy [1,2]. In the following years, saturation biopsy protocols were developed in an effort to further increase the detection rate of CSPC, involving up to 36 cores. However, in biopsy-naïve patients, the detection rate was similar to that of the standard extended (10–12 core) approach, while the morbidity was significantly higher. Therefore, saturation biopsies are currently reserved for selected patients, such as those with persistently elevated PSA levels and prior negative biopsy results [3]. The advent of multiparametric magnetic resonance imaging (mpMRI) has ushered in a new era in prostate imaging and revolutionized biopsy techniques, allowing for both grading of suspect lesions according to cancer risk and accurate lesion targeting [4]. Suspect lesions are graded based on their MRI appearance using the Prostate Imaging Reporting and Data System (PIRADS) score, which predicts the likelihood of significant cancer detection [5,6,7,8]. The PIRADS score ranges from 1, reflecting a very low risk of prostate cancer detection (e.g., less than 5%), to 5, implying a very high risk (e.g., more than 90%) [9]. The decisions to proceed with a prostate biopsy in each patient, or to target a specific lesion, are based on these predictions. However, the accuracy of any test is influenced not only by the test’s performance but also by the target population characteristics. In a previous study on 12-core random prostate biopsies, we demonstrated that the likelihood of a positive biopsy increased 5-fold in small versus large prostates [10]. Several other studies have confirmed this observation, reporting that clinically significant prostate cancer (CSPC) is detected more frequently in smaller prostates as compared with larger ones [11,12,13,14,15]. In the era of random prostate biopsies, these findings could be explained by a dilution effect of a given cancer focus within a larger volume of benign tissue. Alternatively, an observed higher rate of cancer detection in small prostates could stem from an unknown biologic factor intrinsic to small versus large glands [16]. With these considerations in mind, we questioned whether the previously observed higher cancer rate in small prostates remains valid in this era of targeted biopsies. We postulated that when using targeted biopsies, a similar cancer detection rate irrespective of prostate volume would support a dilution effect, while an apparent difference in cancer detection rate could support some biologic difference between small and large prostates. In addition, we questioned whether prostate volume affects the predictive value of the current PIRADS score for clinically significant prostate cancer.

The aim of the current study was to compare CSPC and high-risk prostate cancer (HRPC) detection rates between small and large prostate glands and evaluate the predictive performance of the PIRADS score for CSPC and HRPC in these two groups.

## 2. Materials and Methods

### 2.1. Patients and Methods

Between 30 October 2016 and 13 June 2023, a total of 361 consecutive patients underwent mpMRI-based fusion prostate biopsies at our center. None of them had been previously diagnosed with prostate cancer, and none of them had undergone any previous prostate biopsies. Patients underwent prostate biopsies if there was a clinical suspicion for prostate cancer and an MRI showed any suspect prostate lesion graded PIRADS 3 or higher. Targeted and random biopsies were taken in all patients, and the number of samples was decided at the discretion of the performing surgeon. For the purpose of this study, in patients with multiple MRI lesions, the worst lesion in terms of size and PIRADS grade was considered for analysis. An average of 15 cores was obtained in each procedure (ranging from 4 to 48 biopsy cores). All biopsies were performed under general laryngeal mask anesthesia and a transdermal perineal block. Biopsies were obtained transperineally, using a BK™ (model flex focus 500) ultrasound device (Herlev, Denmark). We used the DK™ fusion system (Ganderkesee, Germany) to create computerized 3-dimensional models of each patient’s prostate and any suspect lesions in it. Prostate MRI exams were obtained in various institutions outside our medical center, as well as from our center. Prostate volume was documented as per the MRI reports, and all suspect prostate lesions were graded by MRI radiologists according to the PIRADS V 2.0 grading system. All biopsy core specimens were processed at our center. The cores were formalin-fixed and paraffin-embedded, as well as sectioned and stained with Hematoxllin-eosin. Pathology reports were written by our institution’s pathologists. Pathology reports were classified as benign versus malignant. Cancers graded Gleason 3 + 3 (ISUP 1) were considered clinically insignificant prostate cancers, whereas any lesions with carcinoma Gleason ≥ 3 + 4 (ISUP ≥ 2) were considered clinically significant prostate cancers (CSPC). High-risk prostate cancers (HRPC) were defined when biopsies showed Gleason ≥ 4 + 4 carcinoma (ISUP 4–5). Demographic and clinical data were abstracted from patients’ charts, including age, serum PSA levels before the biopsy, the presence of firm nodules on digital rectal examination, prostate volume, the highest PIRADS score, the number of biopsy cores, and the pathology report.

The study was approved by the institutional Helsinki Committee in accordance with the principles of the Declaration of Helsinki. Owing to its retrospective design, the requirement for informed consent was waived, and data were collected from electronic medical records.

### 2.2. Statistical Analysis

Prostate volumes and pathology reports showing CSPC were used to define the optimal prostate volume cutoff that would best define the risk for CSPC. All continuous variables were tested for normality using the Kolmogorov–Smirnov (K–S) test. Normally distributed data were presented as mean ± standard deviation (Mean ± SD), whereas non-normally distributed data were reported as median ± interquartile range. For comparisons of continuous variables between groups, independent-samples *t*-tests or Mann–Whitney U tests were employed, as appropriate. Categorical variables were expressed as frequencies (percentages) and compared using chi-square tests. Logistic regression analysis was performed to model the probability of prostate cancer, with results visualized as probability prediction curves with 95% confidence intervals. Receiver-operator curves (ROC) were plotted to evaluate the diagnostic performance of PSA density and prostatic volume across different PIRADS categories, and the area under the ROC curve (AUC) was calculated. The DeLong test was used to compare the curves. MedCalc 23.3 software was used to compare AUC values. Sensitivity, specificity, positive and negative likelihood ratios (+LR and −LR), and the Youden index were also calculated for each diagnostic cutoff. A *p* value < 0.05 was considered indicative of statistical significance. A 44 mL prostate volume cutoff was selected, and the entire patient sample was classified into Group 1 (prostate volume 0–44 mL) and Group 2 (prostate volume > 44 mL). Finally, we used cubic spline models to visualize the relationship between prostate volume, PSA density, and the risk of having prostate cancer. Statistical analyses were performed using SPSS 29.0.

## 3. Results

**Figure 1:**
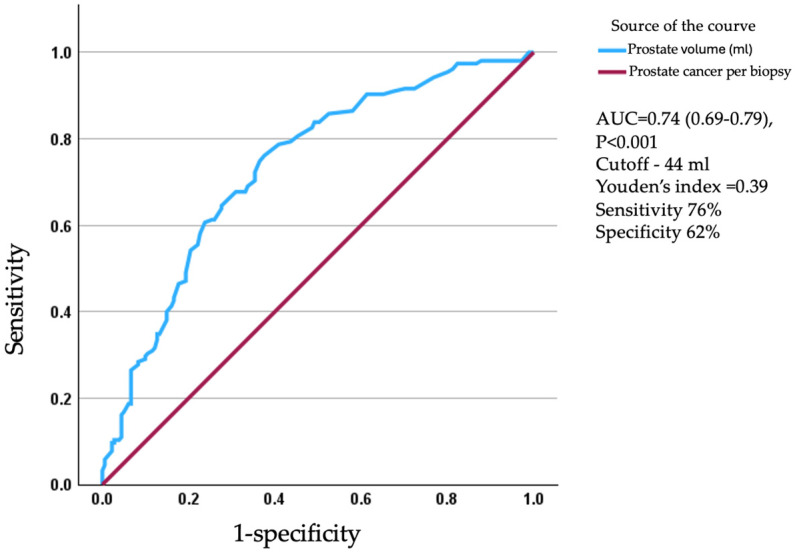
ROC plot designed to determine the best prostate volume cutoff level for any cancer prediction.

A total of 361 consecutive patients who met our inclusion criteria were included in this study; 8 were excluded due to a lack of documentation of prostate volume (Table 1). Based on an ROC plot (AUC = 0.74, 0.69–0.79, Sensitivity 76% Specificity 62%), a prostate volume cutoff of 44 mL was defined to classify the patients into Groups 1 and 2 with prostate volumes below or above this volume cutoff (Figure 1).

Demographic and clinical data on our cohort are shown in Table 1. Serum PSA levels, palpable prostate nodules, and PSA density were all more frequent in Group 1. The distribution of PIRADS grades was not different between groups. The number of both targeted and random biopsy samples was higher in Group 2.

Prostate biopsies positive for any cancer (i.e., ISUP ≥ 1) were detected among 119 (74.3%) patients of Group 1, as compared with 69 (35.5%) in Group 2, *p* < 0.001. However, small prostate volume was also associated with higher cancer severity, as both CSPC and HRPC were significantly more prevalent in Group 1 versus Group 2 (Table 2).

Univariate and multivariable logistic regression analyses showed an association between small prostate volume and any cancer, CSPC, and HRPC (Table 3).

**Figure 2:**
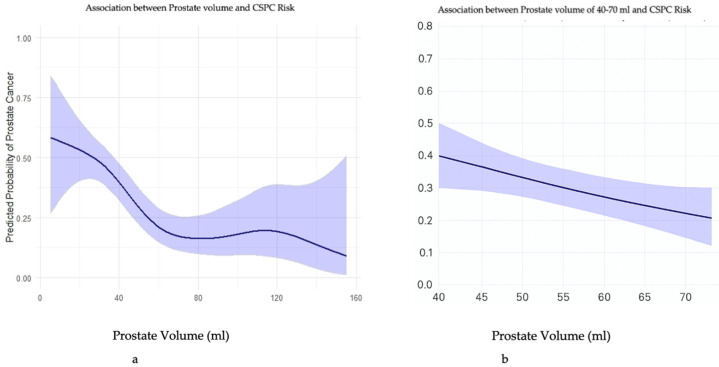
Describes the relationship between the size of the prostate and the risk for prostate cancer. (**a**)—shows the relationship between prostate cancer and prostate size between 20 mL to 160 mL. (**b**)—shows the relationship between prostate cancer and prostate size between 40 mL to 70 mL.

Prostate volume and the risk for CSPC were inversely correlated. For the entire cohort, any 10 mL increase in prostate volume resulted in a 30% decreased risk for PC (Figure 2a). For prostate volumes between 40 and 70 mL (representing 42.8% of this cohort) for every 10 mL increment in prostate volume was associated with a 61% risk reduction for CSPC (OR = 0.939, 95% CI: 0.897–0.983, *p* = 0.008). This association was less prominent for prostates smaller or larger than 40 or 70 mL, respectively (Figure 2b).

Prostate volume significantly affected cancer predictions based on the PIRADS score (Table 4).

Univariate analysis showed that mpMRI prediction of prostate cancer according to PIRADS score showed that lesions with a PIRADS 3 score had a 12.8 risk of CSPC and no risk for HRPC. Lesions with a PIRADS 4–5 score had a 2.8 risk for CSPC and a 2.1 risk for HRPC. In multivariate analysis, the risk in lesions score PIRADS 3 was 16.9 and no risk for HRPC, and, in multivariate analysis, the risk was increased by 4.3 and 3.6, respectively (Table 5).

## 4. Discussion

In this study, we have shown that cancer risk is inversely related to prostate volume. This observation was consistent for the detection of any cancer, CSPC, and HRPC, showing higher cancer detection rates in small versus large prostates. Thus, not only was the likelihood of any cancer detection higher in small versus large prostates, but the aggressiveness of the detected cancers (CSPC and HRPC) was also more prominent in the group with small prostates. This observation, along with the fact that our findings were based on targeted biopsies, alludes to a possible biologic difference between prostates that is volume-dependent, which has an impact on cancer risk, rather than a dilutional effect in large prostate volume. Further genetic and molecular study is required to elucidate the mechanism underlying this effect.

While our results show an inverse and strong impact of prostate volume and the risks of prostate cancer, the volume cutoff specified in this article may be different in other patient samples. Therefore, our main finding is not that the prostate volume cutoff of 44 mL should necessarily be used to distinguish between small and large prostates. Rather, our aim is to show that large prostates tend to have less cancer and a lower risk thereof. External validation and further studies are necessary to corroborate our findings and better define the volume cutoff.

Well-established predictors for a positive prostate biopsy and for clinically significant prostate cancer (CSPC) include serum PSA level, free-to-total PSA ratio, PSA density, the presence of a high PIRADS score lesion on prostate MRI, a positive family history, and genetic predispositions [17,18,19,20,21]. Although the effect of prostate volume is incorporated in the PSA density, we have shown that these two components are independent predictors. In fact, although the mean serum PSA level was higher in Group 2, this group had fewer cancers and less severe cancers than Group 1.

Recent and previous data support our findings [11,12,13,14,21,22,23,24,25,26]; however, all these studies were based on random TRUS-guided prostate biopsies. Uzzo et al. and Karakiewicz et al. demonstrated yields of CSPC of 38% and 39.5%, respectively, in cohorts of up to 2000 patients [22,24]. Our study included only 361 patients, yet the detection rate of 42.5% is similar to their results. All our patients underwent MRI-fusion targeted biopsies, which, according to the previous and recent literature, have a higher yield of CSPC-positive biopsies as compared with random biopsies alone [27]. This observation challenges the notion of a mere “dilution effect” in larger prostates and instead supports the idea of an inherent biological influence of prostate size on tumor grade concordance and aggressiveness. They also showed a linear correlation in a cubic spline model, with a smaller prostate size being associated with CSPC. These findings support our results, as shown in Figure 2a,b. Multivariate analysis demonstrated that smaller prostate volumes were associated with a 5-fold increased risk of detecting clinically significant and high-risk prostate cancer, further supporting a potential biologic effect of prostate size, similar to the findings in other studies [10,11,14,15,22,25,28,29,30]. Some authors have suggested that this phenomenon may be explained by the anatomical distribution of prostatic zones: in larger prostates, the peripheral zone is compressed between the hyperplastic central zone and the prostatic capsule, potentially reducing tumor development or detection in this region [28]. The PCPT and REDUCE trials showed a 25% reduction in prostate volume but an increase in the risk of CSPC [31,32]. Further studies will be necessary to corroborate our results and to define the underlying biological mechanisms responsible for this difference.

In our study, we also observed that with each 1 mL increase in prostate volume, the risk of CSPC decreased by 6.1%, which, to our knowledge, has not been described elsewhere in the literature.

The PI-RADS v2.0 scoring system was developed to estimate the likelihood that a prostate lesion harbors CSPC, thereby guiding decisions regarding biopsy [5,6,7,8,33]. According to our findings, prostate volume has a direct impact on the performance and accuracy of the PI-RADS score. Previous studies have addressed the impact of prostate volume on cancer detection through PSA density calculations [19,20,30,34,35,36]. Given the approximately 20% risk of CSPC in PI-RADS 3 lesions, Drevik et al. demonstrated that an increased PSA density of >0.15 ng/mL^2^ in patients with PI-RADS 3 lesions is associated with a higher likelihood of CSPC; the median prostate volume in their cohort was 58 mL [34]. To date, the use of PSA density as a proxy for prostate volume is not incorporated into the MRI-based PI-RADS scoring system, and there is no radiological parameter explicitly reflecting prostate size beyond PSA density, which indirectly represents either a larger prostate volume or a higher PSA value.

To our knowledge, this is the first report demonstrating that PI-RADS predictive performance is substantially influenced by prostate volume. Incorporating prostate volume into risk stratification could, therefore, refine clinical decision-making around prostate biopsy. Consequently, prostate volume should be considered for inclusion in future iterations of the PI-RADS scoring system. Of particular interest are MRI lesions graded as PI-RADS 3. While lesions graded PI-RADS 4–5 are generally considered to mandate biopsy, the AUA guidelines recommend that clinicians discuss with patients the risks and benefits of biopsy for PI-RADS 3 lesions [37]. Our results show that the risk for CSPC detection in PI-RADS 3 lesions in small prostates is 24%, compared with only 2% for similar lesions in larger glands. Furthermore, in this series, no patient with a PI-RADS 3 lesion in a large prostate was found to have HRPC. Our findings therefore support no role for biopsy in PI-RADS 3 lesions of large prostates. Similarly, while PI-RADS 4–5 lesions predict a high risk for CSPC and HRPC in small prostates, they represent only an intermediate risk in larger glands. Consequently, prostate volume appears to influence significant cancer risk and should therefore be incorporated into the PI-RADS scoring system.

This study is based on a substantial cohort of men undergoing their first prostate biopsy, enhancing the generalizability of our findings. However, some limitations must be acknowledged. This was a retrospective cohort, and therefore, greater variability in patient characteristics may have been present. Magnetic resonance imaging examinations were performed at various institutions, and no central review of imaging data was conducted. Furthermore, while our biopsy protocol included both targeted and systematic biopsies, nearly all CSPC and HRPC cases in this cohort were detected within high-PI-RADS-scoring lesions and were predominantly diagnosed via targeted biopsies, consistent with prior studies [4,9]. Due to the limited sample size, suspicious MRI lesions graded PI-RADS 4 and 5 were analyzed together rather than separately. Further research is needed to address potential differences in CSPC prediction between PI-RADS 4 and 5 lesions in small versus large prostates.

## 5. Conclusions

Prostate volume plays an important role in significant cancer detection, with smaller glands more often affected by more aggressive cancer than large prostates. This effect appears to be an intrinsic prostatic factor and not a sampling error. The PIRADS scoring system performs differently for large versus small prostates, and, as a result, we suggest that a new version of the PIRADS score be designed, accounting for prostate volume. Suspicious PI-RADS 3 lesions in larger prostates may not necessarily require biopsy, although further studies are needed to validate this observation.

## Figures and Tables

**Table 1:** Demographic, imaging, clinical and procedure related characteristics of Study Participants (n = 353) Stratified by Prostate Volume (<44 mL vs. ≥44 mL).

Variable	Prostate Volume by MRI (mL)		*p* Value
	Group 1 (n = 160)	Group 2 (n = 193)	
Age (y)	68.6 ± 7.8	68.5 ± 6.9	0.95
PSA (ng/mL)	5.9 (4.5–9.2)	7.0 (5.4–10.0)	0.002
DRE suspected n (%)	19 (13.0)	9 (4.3)	0.02
Prostate volume (mL)	33.0 (27.0–37.0)	65.5 (54.0–84.0)	-
Max PIRADS			0.15
1–2 n (%)	7 (4.7)	12 (5.7)	
3 n (%)	22 (15.0)	47 (22.7)	
4–5 n (%)	135 (92.4)	131 (63.2)	
Number of samples ROI, n	7.5 ± 3.1	8.8 ± 3.8	<0.001
Number of random samples, n	6.9 ± 2.5	7.6 ± 2.6	0.02
PSA Density (ng/mL^2^)	0.18 (0.13–0.29)	0.11 (0.08–0.15)	<0.001

PSA—prostate specific antigen. DRE—digital rectal examination. PIRADS—Prostate Imaging Reporting and Data System. ROI—region of interest referring to a suspect target on MRI.

**Table 2:** Comparison of targeted and random biopsy results according to prostate volume.

Variable	Prostate Volume by MRI (mL)		*p* Value
	Group 1 (n = 160)	Group 2 (n = 193)	
Any cancer n (%)	119 (74.3)	69 (35.5)	<0.001
Cancer in ROI n (%)	114 (71.2)	62 (32.1)	<0.001
CSPC in ROI n (%)	68 (42.5)	38 (19.6)	<0.001
CSPC in random n (%)	41 (25.6)	15 (7.6)	<0.001
Max ISUP grade group			<0.001
1 n (%)	43 (26.8)	30 (15.5)	
2 n (%)	35 (21.8)	17 (8.6)	
3 n (%)	18 (11.2)	11 (5.6)	
4 n (%)	17 (10.6)	8 (4.0)	
5 n (%)	6 (3.7)	3 (1.5)	

**Table 3:** Univariate and multivariate logistic regression of cancer detection by prostate volume.

	Prostatic Volume (mL) < 44			
	Univariate		Multivariate *	
	OR (95% CI)	*p*	OR (95% CI)	*p*
**All patients**				
Prostatic cancer	5.30 (3.29–8.47)	<0.001	7.31 (4.22–12.7)	<0.001
CSPC	3.50 (2.17–5.65)	<0.001	5.08 (2.87–8.99)	<0.001
High risk PC	2.73 (1.28–5.84)	0.009	4.50 (1.80–11.3)	0.001

* Adjusted for age, DRE, PIRADS and PSA.

**Table 4:** Comparison of Cancer Detection Rates Between Groups by PIRADS Score.

	PIRADS 3			PIRADS 4–5		
	All Cancers	CSPC	HRPC	All Cancers	CSPC	HRPC
Group 1	10	4	1	107	69	21
Group 2	5	1	0	59	37	11
*p*-value	0.003	0.003	0.32	<0.001	<0.001	0.09

CSPC—clinicaly significent prostate cancer; HRPC—high risk prostate cancer.

**Table 5:** Univariate and multivariate analysis of patients with smaller prostates.

End-Point\Predictor	Prostatic Volume (mL) < 44			
	Univariate		Multivariate *	
	OR (95% CI)	*p*	OR (95% CI)	*p*
**PIRADS 3**				
Any cancer	5.55 (1.55–18.8)	0.008	15.3 (2.44–95.5)	0.004
CSPC	12.8 (1.39–118.4)	0.03	16.9 (1.59–178.7)	0.02
High risk PC	-		-	
**PIRADS 4–5**				
Any cancer	5.46 (3.07–9.69)	<0.001	7.63 (4.04–14.4)	<0.001
CSPC	2.83 (1.68–4.77)	<0.001	4.35 (2.34–8.09)	<0.001
High risk PC	2.16 (0.99–4.71)	0.05	3.59 (1.43–9.01)	0.007

* Adjusted for age, DRE, PIRADS and PSA.

## Data Availability

No new data were created or analyzed in this study.

## Abbreviations

The following abbreviations are used in this manuscript:

PI-RADS	Prostate Imaging Reporting and Data System
CSPC	Clinically Significant Prostate Cancer
HRPC	High Risk Prostate Cancer
ROI	Region of Interest
DRE	Digital Rectal Examination
PSA	Prostate Specific Antigen
ISUP	International Society of Urological Pathology
